# Characterization of the genomic region containing the Shadow of Prion Protein (SPRN) gene in sheep

**DOI:** 10.1186/1471-2164-8-138

**Published:** 2007-05-30

**Authors:** Evelyne Lampo, Mario Van Poucke, Karine Hugot, Hélène Hayes, Alex Van Zeveren, Luc J Peelman

**Affiliations:** 1 Department of Nutrition, Genetics and Ethology, Faculty of Veterinary Medicine, Ghent University, B-9820 Merelbeke, Belgium; 2 CRB GADIE, INRA, DGA, Laboratoire de Radiobiologie et d'Etude du Genome, INRA Jouy-en-Josas, F-78352 France ; 1CEA, DSV, DRR, Laboratoire de Radiobiologie et d'Etude du Genome, INRA Jouy-en-Josas, F-78352 France; 3 UR 339 Unité de Génétique biochimique et Cytogénétique, INRA Jouy-en-Josas, F-78352 France

## Abstract

**Background:**

TSEs are a group of fatal neurodegenerative diseases occurring in man and animals. They are caused by prions, alternatively folded forms of the endogenous prion protein, encoded by *PRNP*. Since differences in the sequence of *PRNP *can not explain all variation in TSE susceptibility, there is growing interest in other genes that might have an influence on this susceptibility. One of these genes is *SPRN*, a gene coding for a protein showing remarkable similarities with the prion protein. Until now, *SPRN *has not been described in sheep, a highly relevant species in prion matters.

**Results:**

In order to characterize the genomic region containing *SPRN *in sheep, a BAC mini-contig was built, covering approximately 200,000 bp and containing the genes *ECHS1*, *PAOX*, *MTG1*, *SPRN*, *LOC619207, CYP2E1 *and at least partially *SYCE1*. FISH mapping of the two most exterior BAC clones of the contig positioned this contig on Oari22q24. A fragment of 4,544 bp was also sequenced, covering the entire *SPRN *gene and 1206 bp of the promoter region. In addition, the transcription profile of *SPRN *in 21 tissues was determined by RT-PCR, showing high levels in cerebrum and cerebellum, and low levels in testis, lymph node, jejunum, ileum, colon and rectum.

**Conclusion:**

Annotation of a mini-contig including *SPRN *suggests conserved linkage between Oari22q24 and Hsap10q26. The ovine *SPRN *sequence, described for the first time, shows a high level of homology with the bovine, and to a lesser extent with the human *SPRN *sequence. In addition, transcription profiling in sheep reveals main expression of *SPRN *in brain tissue, as in rat, cow, man and mouse.

## Background

TSEs are a group of fatal neurodegenerative diseases, caused by prions (PrP^Sc^). These infectious particles are alternatively folded forms of the endogenous protein PrP^C^, encoded by *PRNP *[1,2]. Conversion of PrP^C ^into PrP^Sc ^requires the presence of PrP^Sc ^and probably also of a not identified species-specific protein, 'protein X' [3,4].

In sheep, TSE susceptibility is influenced by polymorphisms of the *PRNP *gene, with the alleles coding for alanine, arginine and arginine at positions 136, 154 and 171 of the prion protein associated with a high resistance to classical scrapie and BSE [5]. Nevertheless, this resistance is not absolute, since it has been shown that atypical scrapie can occur in sheep with the genotype ARR/ARR [6-11], sheep with this genotype can be artificially infected with BSE [12] and infectivity has been detected in the spleen of an ARR/ARR sheep, experimentally infected with BSE [13]. Moreover, the presumed resistance of ARR/ARR sheep might be due to a longer incubation period in these animals and subclinically infected sheep might transmit TSE infections unnoticed [14,15]. Therefore, there is growing interest in other genes and proteins which could have an influence on TSE susceptibility in sheep.

One of these genes is *PRND*, a *PRNP *homologue found near the *PRNP *gene and having structural and biochemical similarities with *PRNP*. However, no clear influence of *PRND *on TSE susceptibility has been found to date [16,17]. Also, a number of proteins with a high affinity for the prion protein, among which the 37-kDa/67-kDa laminin receptor, have been discovered [18,19] and could be important as 'protein X' candidates. In addition, gene expression studies in the brain of scrapie-infected mice have identified a large number of genes, potentially involved in the pathogenesis of TSEs [20-22].

Based on comparative genomics, Premzl et al. [23] have discovered *SPRN*, a new candidate gene which codes for the Shadoo protein of 130–150 amino acids. This gene has already been described in man, mouse, rat, fish [23] and cow [24] and is predicted in chimpanzee (GenBank:XM_001146049). The Shadoo protein has also been identified in Sumatran orang-utan, rhesus macaque, white-tufted-ear marmoset, rabbit, guinea pig, dog, little brown bat, gray short-tailed opossum, chicken and western clawed frog [25]. An evolutionary model proposes that *SPRN *shares a common ancestor with *PRNP *[26], since it presents several important similarities with *PRNP*. First, the open reading frame of *SPRN *is located entirely in the last exon, with one preceding non-coding exon (one or two in *PRNP*) [23,27]. In addition, Shadoo is predicted to be extracellular and glycosylphosphatidylinositol-anchored. Moreover, the most remarkable structural feature of Shadoo is the presence of a hydrophobic sequence, composed of aliphatic amino acids and very similar to the hydrophobic sequence typically found in PrP and PrP-like proteins [23,28,29].

Apart from the structural similarities between *SPRN *and *PRNP*, the expression profile of *SPRN *also makes this gene an interesting candidate for further research. According to the results of RT-PCR and Northern blot analyses in cow [24], RT-PCR in rat [23], and cDNA, EST and SAGE map data in man and mouse [25], *SPRN *is mainly expressed in brain tissue, the most important target organ for prion infections. Since PrP knock-out mice [30] and cattle lacking the prion protein [31] show no major phenotypic changes, another gene, possibly *SPRN*, might take over the physiological function of the prion protein.

In this study, the genomic region containing *SPRN *in sheep was investigated using comparative mapping and sequencing and transcription profiling of the *SPRN *gene were performed.

## Results and discussion

### Construction and annotation of a BAC mini-contig containing *SPRN*

**Figure 1:**
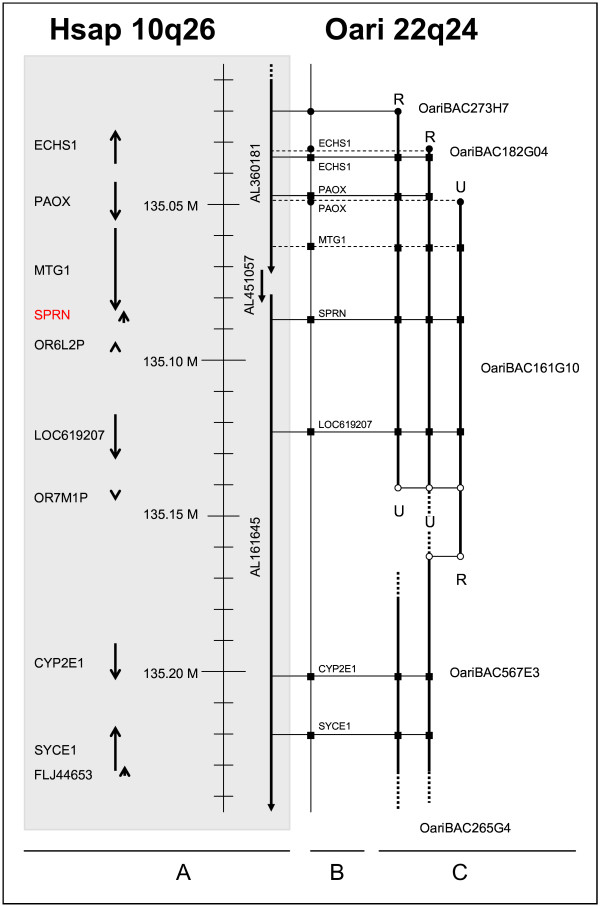
**Comparative mapping of the region containing *SPRN *in man and sheep**. The position and orientation of the human genes and finished HTGs (used to assemble the human genome sequence) are represented in (A) by arrows. The annotated sequences from the BAC contig are shown in a plane map in part B. The sheep BAC contig is drawn in part C. White circles represent BESs used to design primers to construct the contig. Black circles represent annotated BESs. Black squares represent annotated internal BAC sequences. Full lines indicate sequence identity between the sheep sequence and the human sequence and dotted lines sequence identity between the sheep sequence and the bovine orthologue of the human gene on the indicated position. U and R represent respectively the BAC end obtained by the UP and the RP. U and R sides could not be determined for OariBAC265G4 and OariBAC567E3.

The construction of a BAC contig containing *SPRN *was started by screening the INRA ovine BAC library with primers based on the genes *PAOX *and *CYP2E1*, with *PAOX *approximately 30,000 bp proximal from *SPRN *and *CYP2E1 *approximately 120,000 bp distal from *SPRN*, according to the human genome sequence. One BAC clone was found with the *PAOX *primers (OariBAC273H7) and two with the *CYP2E1 *primers (OariBAC265G4 and OariBAC567E3). Primers based on BESs of OariBAC273H7 and OariBAC265G4 did not permit to identify overlapping clones. Therefore, a new screening of the ovine BAC library was performed with the OariBAC273H7 UP primers, resulting in the identification of two new BAC clones (OariBAC182G04 and OariBAC161G10). Primers based on OariBAC161G10 BES (with one primer designed outside the repeat sequence, resulting in an amplicon of which 82 bp are not part of any repetitive element) showed an overlap between the *PAOX *and the *CYP2E1 *subcontig. The total mini-contig thus consists of five BAC clones and is shown in Figure 1. Based on a comparison with the human genome sequence, the contig can be estimated to cover approximately 200,000 bp.

Both BESs and internal BAC sequences were used to annotate the contig. Sequencing of the BESs of the five clones resulted in ten sequences, of which four contain a high amount of repeat sequences (Genbank:EI184567, Genbank:EI184564, Genbank:EI184562 and Genbank:EI184563). Three BESs could be annotated, one (Genbank:EI184569) by comparison with the human genome sequence (Genbank:AL360181), and two (Genbank:EI184565 and Genbank:EI184561) by comparison with bovine genes, showing homology with the intron sequence of respectively *ECHS1 *(Genbank:DQ058603) and *PAOX *(Genbank:DQ058602). Three BESs revealed no relevant homology. Detailed information on the BESs is given in Table 1.

**Table 1:** Characteristics of the BESs.

**OariBAC clone**	**Accession number**	**Length sequence (bp)**	**Annotation (repeats (class/family) or** ** nucleic acid identity with described sequences)**
273H7-UP	EI184560	650	No repeats, no homology found
273H7-RP	EI184569	723	Cow: NM_001076278: 91% Man: AL360181: 91%
567E3-UP	EI184567	752	21–173: L1M4 (LINE/L1) 160–752: BovB (LINE/RTE)
567E3-RP	EI184564	824	29–823: L1MA4A (LINE/L1)
265G4-UP	EI184562	743	114–257: MER34A1 (LTR/ERV1) 318–426: L1MC3 (LINE/L1) 498–688: L1MC3 (LINE1/L1)
265G4-RP	EI184566	454	246–278: TGGG(n) (simple repeat)
182G04-UP	EI184568	171	No repeats, no homology found
182G04-RP	EI184565	391	Cow: DQ058603: 89%
161G10-UP	EI184561	267	Cow: DQ058602: 89%
161G10-RP	EI184563	306	1–210: BovB (LINE/RTE)

Comparison of internal BAC sequences with the human genome sequence revealed the presence of *ECHS1*, *PAOX*, *MTG1*, *SPRN*, *LOC619207*, *CYP2E1 *and at least partial presence of *SYCE1 *in the contig. This is the first time that these genes have been identified in sheep. Due to lack of publicly available sequences in sheep or any related species, no suitable primers could be designed for the genes *OR6L2P *and *OR7M1P*, two genes also present in the human genome sequence corresponding to the sheep contig. Primers based on human sequences were tested for these genes, but no PCR product could be created on sheep DNA. All PCR products show a high level of homology with the human genome sequence, except that of *MTG1*, which shows homology only with the bovine sequence, probably because of the high intron content of the PCR product. Nucleotide identity and amino acid identity and positivity (amino acids are identical or share the same characteristics) of the amplicons with known sequences are shown in Table 2.

**Table 2:** Amplicon characteristics of primers used for the annotation of the contig and the transcription profiling.

**Gene symbol or primer's name**	**Forward primer (5'-3')** **Reverse primer (5'-3')**	**Ta (°C)** ** Length (bp)**	**Accession number amplicons**	**% nucleic acid identity/amino acid identity/amino acid positivity with described sequences**
ECHS1	GCAAAGAATGGGAAAGAACAGCA GGCTCAAAAACCCGCCAGA	65 646	EF215853	OariEST (CD288818): 96/93/94 BtauECHS1 (DQ058603): 96/87/94 HsapECHS1 (NM_004092): 86/80/92
PAOX	GCATCTGGACACCTTCTTTGA GTCCTCCCACACCACCTG	65 274	EF215855	OariEST (DY499183): 99/100/100 BtauPAOX (DQ058602): 95/98/100 HsapPAOX (NM_152911): 90/88/94
MTG1	CAGCTACCGCTATCACCGAGGA GCGAGGACTTGCCCACGTT	60 154	EF215854	BtauMTG1 (DQ058604): 92/75/85
SPRN	GCGAGGGTGCGTGTGAGG CCTGAGGTCCACGCCCAGTA	68 236	-	BtauSPRN (DQ058606): 95/92/93 HsapSPRN (NM_001012508): 79/76/80
LOC619207	GGCTGGTCAACGGCAGCA GGCTCTGTCCCCACGCAGT	65 220	EF215856	HsapLOC619207 (NT_017795): 88/73/84
CYP2E1	AAGAAATTGACAGGGTGATTGG AGGGAAGGAGGTCGATGAAT	60 117	EF215857	OariEST (EE790798): 98/100/100 BtauCYP2E1 (DQ058608): 98/97/100 HsapCYP2E1 (NM_000773): 91/86/94
SYCE1	GACAGCGGCAAGGAGCAGTT GCTTCACATCCTCCAGCTTTGC	65 149	EF215858	BtauSYCE1(NM_001038149): 100/100/100 HsapSYCE1 (NT_017795): 90/90/90
OariBAC273H7 UP	GGGACCATCCTGCTGTGACG TCCACTGTCTGCGTCGTCCTC	65 328	EI184560	-
OariBAC265G4 RP	TGAGAGGTAAGAAGACCACCAAA TCAACCGCAGAACTATGAACC	63 287	EI184566	-
OariBAC161G10 RP	CAGCCTTGACGCACTCCTTT TTTAGAACTGGGCCACACAGC	63 200	EI184563	-
ACTB	CGCAGACAGGATGCAGAAAGA GCTGATCCACATCTGCTGGAA	60 148	DQ386889	-
UP	CGACGTTGTAAAACGACGGCCAG	55 -	-	-
RP	CACAGGAAACAGCTATGACCATGATTACG	55 -	-	-

For amplicons containing both exon and intron sequences (*ECHS1*, *MTG1 *and *SYCE1*), sequence identity and positivity is shown for the exon parts of the amplicon.

Order as well as orientation of the identified genes present in the sheep contig are identical to those of the corresponding region in man (Hsap10q26; human genome sequence), supporting conserved linkage between these species. In cattle, *PAOX *has an opposite orientation (Btau26q23; [24]). Remarkably, the block *ECHS1*, *PAOX*, *MTG1 *and *SPRN *seems highly conserved, as it is also found in man, mouse and even fugu [23]. The scavenger receptor close to *SPRN *in man and mouse, mentioned by Premzl et al. [23], is probably *LOC619207 *(as it codes for a scavenger receptor protein family member), and is therefore also present in the sheep contig.

### FISH mapping of the contig

**Figure 2:**
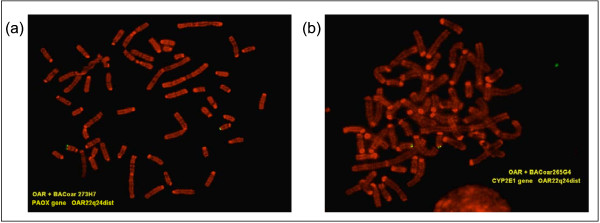
**FISH localization of OariBAC273H7 and OariBAC265G4**. (a) FISH map of OariBAC273H7 and (b) FISH map of OariBAC265G4.

FISH mapping positioned clones OariBAC273H7 and OariBAC265G4 on chromosome Oari22q24 (Figures 2a and 2b). These results are in accordance with the heterologous chromosome painting data reported by Iannuzzi et al., showing correspondences between the distal part of human chromosome 10 and sheep chromosome 22 [32]. Moreover, the presence of OariBAC273H7 and OariBAC265G4, the most exterior BAC clones of the contig, on the same chromosome location, shows that no chromosome jumping has taken place during the construction of the contig. Localization of the contig maps the genes *ECHS1*, *PAOX*, *MTG1*, *SPRN*, *LOC619207*, *CYP2E1 *and *SYCE1 *to Oari22q24.

### Sequencing *SPRN *in sheep

Sequencing of the *SPRN *gene was started with primers based on the bovine sequence NW_993476 (a contig containing *SPRN *partially). Next, primers based on the sheep *SPRN *sequence obtained during this work and primers based on the bovine sequence DQ058606 were used (Table 3). The overlapping amplicons resulted in a sequence of 4,544 bp, covering the entire *SPRN *gene and a stretch of 1206 bp of the promoter region (Genbank:DQ870545). Exon 1 has a length of 108 bp, intron 1 a length of 735 bp and exon 2 a length of 2495 bp, of which 438 bp are coding sequence. All overlaps between the different amplicons (average overlap: 172 bp) were 100% identical. The intron/exon splice sites and the position of the coding sequence were determined by comparison with the bovine (Genbank:DQ058606) and human (Genbank:NW_001012508) *SPRN *sequences. The 3' end of the gene was determined by sequencing cDNA from cerebrum mRNA.

**Table 3:** Amplicon characteristics of the *SPRN *primers used.

**Number *SPRN *primers**	**Forward primer (5'-3')** **Reverse primer (5'-3')**	**Ta (°C)** **Length (bp)**	**Position in sequence DQ870545**
1	ACTCCGGCTCTGGGCTCTGT GGCTCTGTCTTGCTTTCCAAGGT	63 645	1–645
2	TTCAGGGACCACAGGATCGAA CCACGGGCTTCAGCACCTC	60 487	549–1035
3	GTGCGAAGTTGGGGTGAGGA AGCGGGTGAGGGTCTGGAAG	60 330	973–1302
4	GAGGACGGATGCGGTGGAG CCAAAGGAAGCGGGTGAGG	64 601	988–1588
5	CAGGGGTCGCCTCTGGTC CTGCTGGAGGAGTGGGGAGT	64 507	1375–1881
6	ACCCTCACCCGCTTCCTTTG TAGCAGCAGAGCCCAGCACA	66 486	1619–2104
7	CCGCCCCTGAGCCCTGAC CCGCATCCTCCAGGCCAAG	66 334	1967–2300
8	CCGTGTGCTGGGCTCTGCTG GGCGCTCCGTCCTCTGCATC	68 256	2082–2337
9	GCGAGGGTGCGTGTGAGG CCTGAGGTCCACGCCCAGTA	68 236	2189–2424
10	AGCTTGGCCCGGAGGATG CCTGGGTGAGGGTGTTCTGG	66 678	2384–3061
11	AGCCCACCCTGGACACTTGA AGCTGGAGGGAAAAGCACCTG	66 446	2852–3297
12	GGTGCGTCTGTGGATCTGTGAG CTCCCGCTGGTCTTGTGGAG	66 655	3071–3725
13	TCTGGTTGCGGTCAGGGTCT TGAAGTCGGGTTGTTGAGTGGAA	65 423	3574–3996
14	GCCAGATGCCCTCCATCCTC CTGCGAGCACCTTCCAGCTAA	65 571	3760–4330
15	GGAGGGTCGCAACACCACT GGCAGCAGAGTTTATTTCACCAC	65 279	4226–4504
16	CCCGCTTCCAGAATGTGCAG TCCCAGTTCCGTCATGGTCGT	66 300	4263–4544
17	GTGACCTTCCTGCCCTTCAGGTGT GTGTCTGCCCTTCAGCTTCGTGA	68 250	4354–4544
18	TCCCAGTTCCGTCATGGTCGTGTCTGCCCTTCAGCTTCGTGA(T)_18_	-	-

The coding sequence of sheep *SPRN *has 93% respectively 78% nucleic acid identity, and 95% respectively 76% amino acid identity with cow and man. The complete sequence obtained here (Genbank:DQ870545) has 92% nucleic acid identity with the bovine *SPRN *sequence (Genbank:DQ058606). The GC content of the coding sequence in sheep is high (79%), as in cow (77%) and man (79%). The overall GC content of the obtained *SPRN *sequence in sheep is 70%.

**Figure 3:**
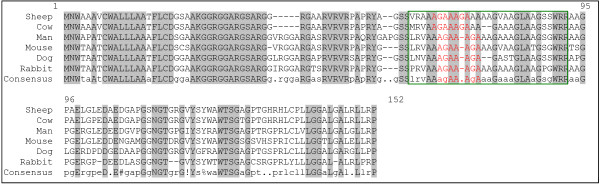
**Comparison of the SPRN amino acid sequence in sheep and other mammals**. The sequences used are sheep: Genbank:DQ870545, cow: Genbank:DQ058606, man: Genbank:NM_001012508, mouse: Genbank:NM_183147, dog: Genbank:BN000838 and rabbit: Genbank:BN000843. Grey indicates conservation between the six species. The hydrophobic region is indicated with a green rectangle, the palindrome sequence is indicated in red.

Comparison of the deduced amino acid sequence between sheep and other mammals reveals a high level of conservation in the typical hydrophobic region of *SPRN *(see Figure 3). This hydrophobic sequence, containing the palindrome sequence AGAAAGA, is a typical characteristic of *SPRN *and is very similar to the hydrophobic region found in PrP and PrP-like proteins [23,28,29]. In prion protein, this region has remained highly conserved during evolution [27,33] and thus may be an important functional region. The synthetic peptide PrP 106–126, containing the hydrophobic sequence, has been shown neurotoxic *in vitro *and *in vivo *[34,35] and this neurotoxicity seems to depend on the presence of the palindrome sequence [36]. The hydrophobic region also plays a role in the conversion of PrP^C ^into PrP^Sc^. PrP^C ^without the palindrome sequence can not be converted into PrP^Sc ^[37] and does not bind with PrP^Sc ^or PrP 106–126 [38]. Moreover, a PrP transgene expressing a mutant PrP with a deletion of the palindrome region acts as a dominant-negative mutant, inhibiting the conversion of wild-type PrP^C ^into PrP^Sc ^[38].

### Transcription profiling of *SPRN *by RT-PCR

**Figure 4:**
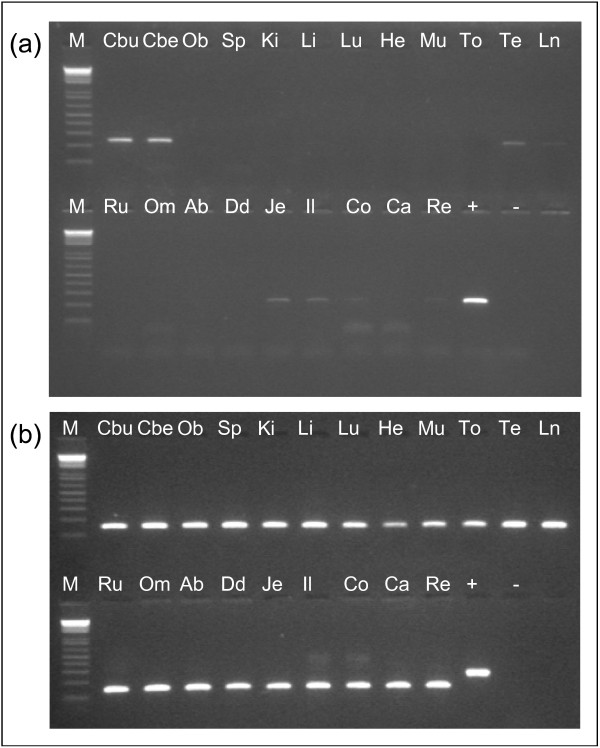
**Transcription profiling of *SPRN *in 21 sheep tissues**. (a) RT-PCR with *SPRN *primers no. 9 and (b) Control PCR with *ACTB *primers. Marker (M) is the 1 Kb+ ladder (Invitrogen). Samples are cDNA of cerebrum (Cbu), cerebellum (Cbe), obex (Ob), spleen (Sp), kidney (Ki), liver (Li), lung (Lu), heart (He), muscle (Mu), tongue (To), testis (Te), lymph node (Ln), rumen (Ru), omasum (Om), abomasum (Ab), duodenum (Dd), jejunum (Je), ileum (Il), colon (Co), caecum (Ca), rectum (Re), positive control (+) and negative control (-).

After isolation of total RNA and subsequent conversion to cDNA, the presence of *SPRN *transcripts was examined by RT-PCR with *SPRN *primer no. 9 in 21 different tissues. The RT-PCR, performed in triplicate, showed high levels of *SPRN *mRNA in cerebrum and cerebellum (Figure 4a) and low levels in testis, lymph node, jejunum, ileum, colon and rectum (very faint bands on Figure 4a). No *SPRN *mRNA was detected in obex, spleen, kidney, liver, lung, heart, muscle, tongue, rumen, omasum, abomasum, duodenum and caecum. An RT-PCR with *SPRN *primer no. 11, also performed in triplicate, on the same tissues showed similar patterns. The results of a control PCR with *ACTB *primers are shown in Figure 4b.

These results of the transcription profiling in sheep are in good agreement with the data available in other species. Results of RT-PCR and Northern blot analyses in cattle [24], RT-PCR in rat [23] and cDNA, EST and SAGE map data analyses in man and mouse [25] all show that expression of *SPRN *is highest in brain tissue. Comparison of the *SPRN *transcription profile of the non-brain tissues between sheep and other species is more difficult, as the transcription level is lower in the other positive tissues, in sheep as well as in cow and rat.

Transcription profiling was performed by RT-PCR. This method permits the rapid testing of a large number of different tissues for the presence of a certain transcript. RT-PCR does not give detailed quantitative information on expression, therefore more time consuming methods like real-time PCR or real competitive PCR are needed [39]. However, RT-PCR results give an overall view and can be the basis to choose tissues of interest for more extended, quantitative experiments.

## Conclusion

In this study, *SPRN *as well as six genes surrounding the *SPRN *locus, *ECHS1*,*PAOX*,*MTG1*,*LOC619207*,*CYP2E1 *and *SYCE1*, have been identified in sheep for the first time. A contig containing these genes was constructed and annotated, suggesting conserved linkage between sheep and man in this region. The contig was FISH mapped to Oari22q24. A 4,544 bp fragment was also sequenced, covering the entire *SPRN *gene and 1206 bp of the promoter region. A high level of sequence homology was found with the bovine *SPRN *and, to a lesser extent, with the human *SPRN*. In addition, the transcription profile of *SPRN *was determined in 21 ovine tissues, confirming that *SPRN *is mainly expressed in brain tissue. These results are the first description of the *SPRN *gene in sheep and should be useful as a basis for further research on this prion-like protein.

## Methods

### Primer design and PCR

Primers were designed with Primer3 [40] based on sequences found in NCBI Entrez Gene [41] or on BESs, and all amplicons were verified by sequencing. A list of the primers used with their conditions is given in Tables 2 and 3. PCR was performed with 0.5 U Faststart Taq DNA Polymerase (Roche), 2.0 mM Mg and 200 μM (each) dNTPs (Bioline) on 200 ng BAC DNA, 20–200 ng RNA or on reverse transcribed RNA. For the amplification of *SPRN *sequences, a 5x solution of GC-rich (supplied with the Faststart Taq DNA Polymerase) was added. PCR conditions were 5 min at 95°C, 40 cycles of 30 s at 95°C, 30 s at the annealing temperature and 1 min at 72°C, and a final 10 min elongation at 72°C.

### Construction and annotation of a BAC contig

Primers for the genes *PAOX *and *CYP2E1 *were used for the initial screening of the INRA ovine BAC library by PCR [42]. BAC DNA from three isolated BAC clones was purified from 200 ml culture using the Qiagen Plasmid Midi kit (Qiagen) and the BAC ends were sequenced with UP and RP, using 1 μg DNA per reaction. Primers designed on the BESs of the isolated BAC clones were used to find overlaps between these BAC clones and the OariBAC273H7 UP primers were used to screen the INRA ovine BAC library for new BAC clones in order to close the gap between the two subcontigs. Annotation of the contig was performed by comparing BESs with the human genome sequence and by PCR with primers for genes presumed to be present in the contig. Comparisons were done with NCBI Blast [43] and repeat sequences were detected and identified with Repeatmasker Web Server [44].

### FISH

For probe preparation, BAC DNA extracts were prepared according to standard protocols and purified with the S.N.A.P. K1900-01 Miniprep kit (Invitrogen life technologies). DNA was then labelled by nick-translation with biotin-14-dATP (BioNick 18247-015 labelling system, Invitrogen life technologies), mixed with 100x total sonicated herring sperm DNA and 100x total sonicated sheep DNA, ethanol precipitated, slightly dried and resuspended in hybridization buffer.

R-banded chromosome spreads were obtained from sheep embryo fibroblast cell cultures synchronized with an excess of thymidine and treated with 5-bromodeoxyuridine during the second half of S phase [45]. Fluorescent in situ hybridization, signal detection and R-banding were performed as previously described [46] with 50–100 ng of biotin-14-dATP labelled probe per slide. Before hybridization to the chromosomes, probes were denatured at 100°C for 10 min and pre-hybridized at 37°C for 30–60 min. Slides were examined under a Zeiss Axioplan 2 epifluorescence microscope and the Applied Imaging Cytovision (version 2.7) software was used for image capturing and analysis. Chromosome identification and band nomenclature for sheep chromosomes follow the R-banded standard ideogram reported in ISCNDB2000 [47].

### Sequencing

For sequencing *SPRN *in sheep, the primers mentioned in Table 3 were used. Amplicons of the *SPRN *primers no. 2, 3, 4 and 5 were cloned and sequenced with UP and RP. The other amplicons were sequenced by direct sequencing.

The 3' end of the ovine *SPRN *sequence was obtained using mRNA from cerebrum tissue, isolated with the Illustra™ Quickprep Micro mRNA Purification kit according to the manufacturer's protocol. The obtained mRNA then was converted into cDNA with the Improm-II Reverse Transcriptase kit (Promega) using a newly designed oligo dT primer (*SPRN *primer no. 18) which adds 42 bp to the cDNA. Finally, a PCR with *SPRN *primers no. 16, followed by a PCR with the nested *SPRN *primers no. 17 (both creating an amplicon including the polyA sequence) was performed on 10x diluted cDNA and the obtained amplicon was directly sequenced with *SPRN *primers no. 17.

All sequencing was performed on a Applied Biosystems 3730xl DNA Analyser with the BigDye Terminator v3.1 Cycle Sequencing Kit (Applied Biosystems).

### RNA isolation, cDNA synthesis and RT-PCR

Tissues for RNA isolation were collected in a commercial sheep slaughterhouse, immediately frozen in liquid nitrogen, crushed to powder the same day and stored at -80°C. Total RNA was isolated with the Rneasy plus mini kit (Qiagen) on 30 mg tissue, except for heart, muscle and tongue, where TRIR (ABgene) and 80–100 mg tissue were used. Both methods were performed according to the manufacturer's protocol and followed by a DNase treatment with RQ1 RNase-free DNase (Promega) and a spin-column purification with Microcon YM-100 (Millipore), according to the product's user guides. RNA concentration and OD_260/280 _ratio of the samples were measured with the Nanodrop ND-1000 Spectrophotometer (Isogen) and RNA quality was measured by evaluation of the 28S and the 18S ribosomal bands on a 0.8% agarose gel. Also, a minus RT-PCR was performed on 1 μl RNA to confirm the absence of any DNA contamination. After RNA controls, 0.2–1 μg RNA was converted into cDNA with the Improm-II Reverse Transcriptase kit (Promega) using Random and Oligo dT primers (each 0.5 μg per reaction), and the conversion was confirmed by a PCR with *ACTB *primers (giving an amplicon of different length on gDNA and cDNA) on 10x diluted cDNA. Determination of the transcription profile of *SPRN *was performed with *SPRN *primers no. 9 and *SPRN *primers no. 11 on 10x diluted cDNA.

## List of abbreviations

*ACTB*: gene coding for actin-beta

ARR: genotype of *PRNP *coding for alanine-arginine-arginine at positions 136, 154 and 171 of PrP

BAC: bacterial artificial chromosome

BES: BAC end sequence

BSE: bovine spongiform encephalopathy

Btau: *Bos taurus*

cDNA: complementary DNA

*CYP2E1*: gene coding for cytochrome P450, family 2, subfamily E, polypeptide 1

*ECHS1*: gene coding for enoyl Coenzyme A hydratase, short chain, 1, mitochondrial

ERV: endogenous retrovirus

EST: expressed sequence tag

FISH: fluorescence in situ hybridization

gDNA: genomic DNA

HTG: high throughput genomic sequence

Hsap: *Homo sapiens*

LINE: long interspersed nuclear element

*LOC619207*: gene coding for scavenger receptor protein family member

LTR: long terminal repeat

*MTG1*: gene coding for mitochondrial GTPase 1 homolog (S. cerevisiae)

Oari: *Ovis aries*

*PAOX*: gene coding for polyamine oxidase (exo-N4-amino)

PCR: polymerase chain reaction

*PRND*: gene coding for prion protein 2 (dublet)

*PRNP*: gene coding for prion protein

PrP: prion protein

PrP^C^: cellular form of the prion protein

PrP^Sc^: disease causing form of the prion protein

RP: reverse primer

RT: reverse transcriptase

SAGE: serial analysis of gene expression

*SPRN*: gene coding for Shadow of prion protein

*SYCE1*: gene coding for synaptonemal complex central element protein 1

TSE: transmissible spongiform encephalopathy

UP: universal primer

## Authors' contributions

EL carried out the contig building and annotation, the sequencing, the transcription profiling, and drafted this manuscript. MVP participated in the study design and provided experimental support. KH performed the BAC screening. HH supervised the FISH mapping. AVZ supervised the study. LJP participated in the study design and also supervised the study. All authors read and approved the final manuscript.
